# Supplementary material for: Efficacy of Combined Oral Isotretinoin and Desloratadine or Levocetirizine vs. Isotretinoin Monotherapy in Treating Acne Vulgaris: A Systematic Review and Meta-Analysis of Randomized Controlled Trials
Source: Biomedicines. 2025 Jul 30;13(8):1847. doi: 10.3390/biomedicines13081847 (PMC12383536; doi:10.3390/biomedicines13081847)
Supplement: Supplementary file 1 [file biomedicines-13-01847-s001.zip › biomedicines-3760579-supplementary.pdf]

Research strategy:

Pubmed (Medline) and Cochrane:

(Acne OR “Acne vulgaris”) AND (Antihistamine OR Desloratadine OR Levocetirizine OR H1 antagonist OR “H1 receptor blocker” OR “H1 blocker”) AND (Isotretinoin OR “13-cis retinoic acid”)

Embase:

('acne'/exp OR acne OR 'acne vulgaris'/exp OR 'acne vulgaris') AND ('antihistamine'/exp OR antihistamine OR 'desloratadine'/exp OR desloratadine OR ‘levocetirizine’/exp OR levocetirizine OR 'h1 antagonist'/exp OR 'h1 antagonist' OR (('h1'/exp OR h1) AND ('antagonist'/exp OR antagonist)) OR 'h1 receptor blocker'/exp OR 'h1 receptor blocker' OR 'h1 blocker'/exp OR 'h1 blocker') AND ('isotretinoin'/exp OR isotretinoin OR '13-cis retinoic acid'/exp OR '13-cis retinoic acid')

	Isotretinoin + Antihistamine			Isotretinoin				Mean difference
Study or Subgroup	Mean	SD	Total	Mean	SD	Total	Weight	IV, Random, 95% CI
**1.1.1 Desloratadine**								
El-Ghareeb 2025	6.43	8.1	28	6.37	8.15	30	12.1%	0.06 [-4.12 , 4.24]
Elsekily 2024	10.5	5.95	20	9.5	5.52	10	11.7%	1.00 [-3.30 , 5.30]
Hazarika 2024	2.24	8.45	41	2.24	9.75	30	11.6%	0.00 [-4.34 , 4.34]
Lee 2014	12.61	7.92	20	5.69	6.91	20	10.8%	6.92 [2.31 , 11.53]
Van 2019	5.79	5.24	31	3.42	5.59	31	18.2%	2.37 [-0.33 , 5.07]
**Subtotal (Wald[a])**			**140**			**121**	**64.5%**	**2.02 [-0.06 , 4.10]**
Test for overall effect: Z = 1.91 (P = 0.06)								
Heterogeneity: Tau² (REML[b], 95% CI) = 1.64 [0.00 , 63.00]; Chi² = 6.30, df = 4 (P = 0.18); I² = 29%								
**1.1.2 Levocetirizine**								
Elsekily 2024	8	6.02	20	9.5	5.52	10	11.7%	-1.50 [-5.82 , 2.82]
Pandey 2019	10.44	3.86	50	6.35	4.3	50	23.8%	4.09 [2.49 , 5.69]
**Subtotal (Wald[a])**			**70**			**60**	**35.5%**	**1.67 [-3.76 , 7.10]**
Test for overall effect: Z = 0.60 (P = 0.55)								
Heterogeneity: Tau² (REML[b], 95% CI) = 12.86 [0.35 , >128.60624000000004]; Chi² = 5.65, df = 1 (P = 0.02); I² = 82%								
**Total (Wald[a])**			**210**			**181**	**100.0%**	**2.10 [0.18 , 4.03]**
Test for overall effect: Z = 2.14 (P = 0.03)								
Test for subgroup differences: Chi² = 0.01, df = 1 (P = 0.91), I² = 0%								
Heterogeneity: Tau² (REML[b], 95% CI) = 3.38 [0.00 , 35.36]; Chi² = 13.38, df = 6 (P = 0.04); I² = 55%								

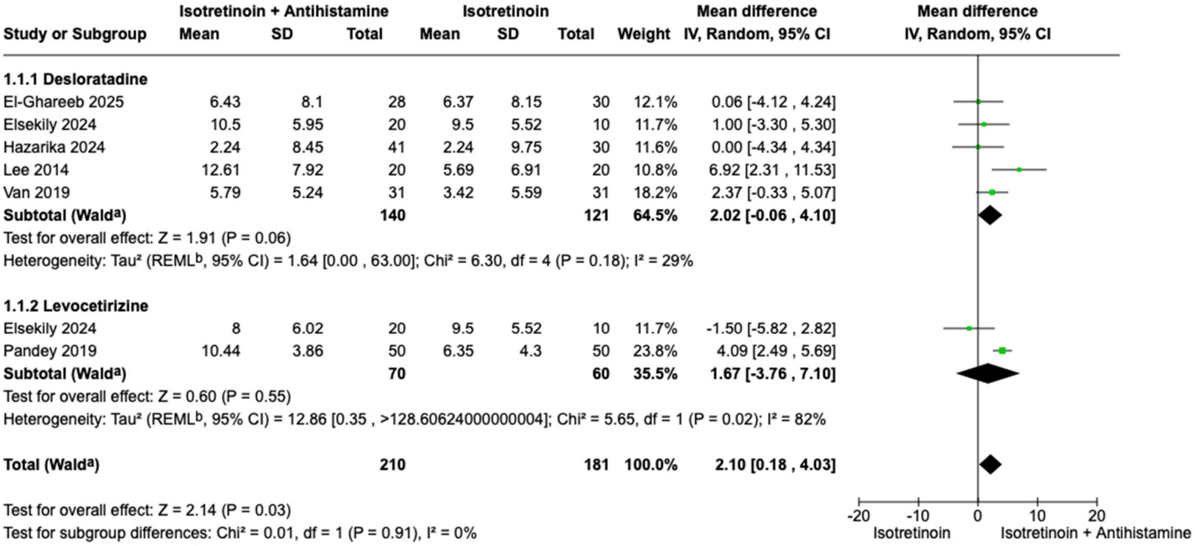

Footnotes
[a]CI calculated by Wald-type method.
[b]Tau² calculated by Restricted Maximum-Likelihood method.

Figure S1. GAGS mean change from baseline till week 4

Study or Subgroup	Isotretinoin + Antihistamine Mean	SD	Total	Isotretinoin Mean	SD	Total	Weight	Mean difference IV, Random, 95% CI
**1.1.1 Desloratadine**								
✓ El-Ghareeb 2025	6.43	8.1	28	6.37	8.15	30	13.1%	0.06 [-4.12 , 4.24]
✓ Elsekily 2024	10.5	5.95	20	9.5	5.52	10	12.6%	1.00 [-3.30 , 5.30]
✓ Hazarika 2024	2.24	8.45	41	2.24	9.75	30	12.5%	0.00 [-4.34 , 4.34]
✕ Lee 2014	12.61	7.92	20	5.69	6.91	20	0.0%	6.92 [2.31 , 11.53]
✓ Van 2019	5.79	5.24	31	3.42	5.59	31	20.8%	2.37 [-0.33 , 5.07]
**Subtotal (Wald[a])**			**120**			**101**	**59.0%**	**1.27 [-0.55 , 3.09]**
Test for overall effect: Z = 1.37 (P = 0.17)								
Heterogeneity: Tau^2 (REML[b], 95% CI) = 0.00 [0.00 , 14.11]; Chi^2 = 1.30, df = 3 (P = 0.73); I^2 = 0%								
**1.1.2 Levocetirizine**								
✓ Elsekily 2024	8	6.02	20	9.5	5.52	10	12.6%	-1.50 [-5.82 , 2.82]
✓ Pandey 2019	10.44	3.86	50	6.35	4.3	50	28.5%	4.09 [2.49 , 5.69]
**Subtotal (Wald[a])**			**70**			**60**	**41.0%**	**1.67 [-3.76 , 7.10]**
Test for overall effect: Z = 0.60 (P = 0.55)								
Heterogeneity: Tau^2 (REML[b], 95% CI) = 12.86 [0.35 , >128.60624000000004]; Chi^2 = 5.65, df = 1 (P = 0.02); I^2 = 82%								
**Total (Wald[a])**			**190**			**161**	**100.0%**	**1.60 [-0.30 , 3.51]**

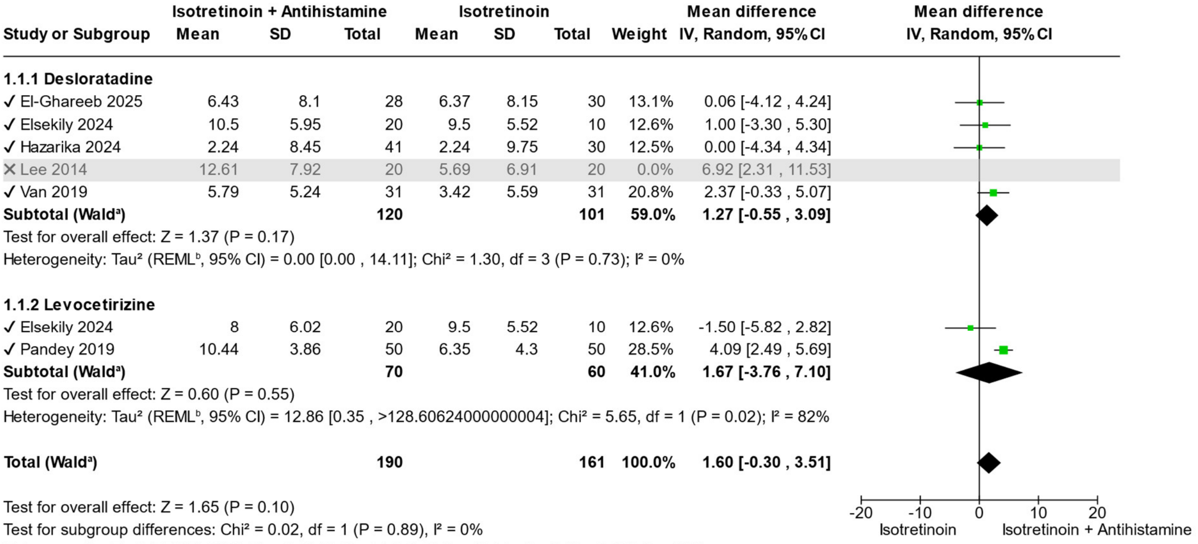

Test for overall effect: Z = 1.65 (P = 0.10)
Test for subgroup differences: Chi^2 = 0.02, df = 1 (P = 0.89), I^2 = 0%
Heterogeneity: Tau^2 (REML[b], 95% CI) = 2.65 [0.00 , 21.07]; Chi^2 = 10.13, df = 5 (P = 0.07); I^2 = 50%

Footnotes
[a]CI calculated by Wald-type method.
[b]Tau^2 calculated by Restricted Maximum-Likelihood method.

Figure S2. GAGS mean change from baseline till week 4

Study or Subgroup	Isotretinoin + Antihistamine Mean	SD	Total	Isotretinoin Mean	SD	Total	Weight	Mean difference IV, Random, 95% CI
**1.1.1 Desloratadine**								
✓ El-Ghareeb 2025	6.43	8.1	28	6.37	8.15	30	15.4%	0.06 [-4.12 , 4.24]
✓ Elsekily 2024	10.5	5.95	20	9.5	5.52	10	15.0%	1.00 [-3.30 , 5.30]
✓ Hazarika 2024	2.24	8.45	41	2.24	9.75	30	14.9%	0.00 [-4.34 , 4.34]
✓ Lee 2014	12.61	7.92	20	5.69	6.91	20	14.0%	6.92 [2.31 , 11.53]
✕ Van 2019	5.79	5.24	31	3.42	5.59	31	0.0%	2.37 [-0.33 , 5.07]
**Subtotal (Wald[a])**			**109**			**90**	**59.4%**	**1.91 [-1.23 , 5.04]**
Test for overall effect: Z = 1.19 (P = 0.23)								
Heterogeneity: Tau^2 (REML[b], 95% CI) = 5.29 [0.00 , >100]; Chi^2 = 6.20, df = 3 (P = 0.10); I^2 = 52%								
**1.1.2 Levocetirizine**								
✓ Elsekily 2024	8	6.02	20	9.5	5.52	10	15.0%	-1.50 [-5.82 , 2.82]
✓ Pandey 2019	10.44	3.86	50	6.35	4.3	50	25.6%	4.09 [2.49 , 5.69]
**Subtotal (Wald[a])**			**70**			**60**	**40.6%**	**1.67 [-3.76 , 7.10]**
Test for overall effect: Z = 0.60 (P = 0.55)								
Heterogeneity: Tau^2 (REML[b], 95% CI) = 12.86 [0.35 , >128.60624000000004]; Chi^2 = 5.65, df = 1 (P = 0.02); I^2 = 82%								
**Total (Wald[a])**			**179**			**150**	**100.0%**	**1.95 [-0.45 , 4.36]**

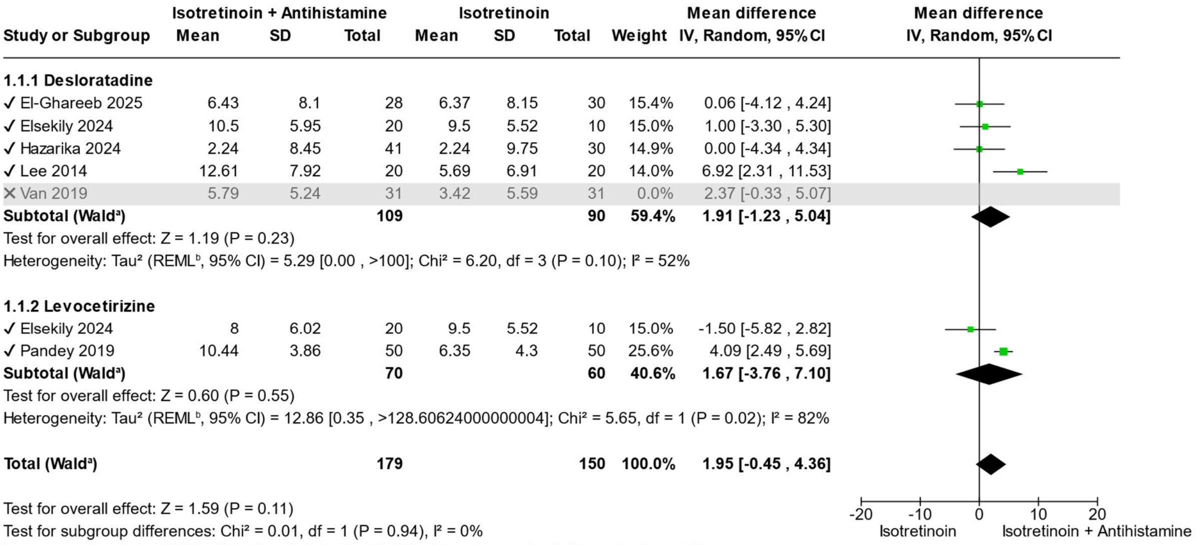

Test for overall effect: Z = 1.59 (P = 0.11)
Test for subgroup differences: Chi^2 = 0.01, df = 1 (P = 0.94), I^2 = 0%
Heterogeneity: Tau^2 (REML[b], 95% CI) = 5.23 [0.08 , 54.54]; Chi^2 = 13.26, df = 5 (P = 0.02); I^2 = 62%

Footnotes
[a]CI calculated by Wald-type method.
[b]Tau^2 calculated by Restricted Maximum-Likelihood method.

Figure S3. GAGS mean change from baseline till week 4

Study or Subgroup	Isotretinoin + Antihistamine Mean	SD	Total	Isotretinoin Mean	SD	Total	Weight	Mean difference IV, Random, 95% CI
**1.1.1 Desloratadine**								
✓ El-Ghareeb 2025	6.43	8.1	28	6.37	8.15	30	15.7%	0.06 [-4.12 , 4.24]
✓ Elsekily 2024	10.5	5.95	20	9.5	5.52	10	15.2%	1.00 [-3.30 , 5.30]
✓ Hazarika 2024	2.24	8.45	41	2.24	9.75	30	15.0%	0.00 [-4.34 , 4.34]
✓ Lee 2014	12.61	7.92	20	5.69	6.91	20	13.9%	6.92 [2.31 , 11.53]
✓ Van 2019	5.79	5.24	31	3.42	5.59	31	25.1%	2.37 [-0.33 , 5.07]
**Subtotal (Wald[a])**			**140**			**121**	**84.9%**	**2.02 [-0.06 , 4.10]**
Test for overall effect: Z = 1.91 (P = 0.06)								
Heterogeneity: Tau² (REML[b], 95% CI) = 1.64 [0.00 , 63.00]; Chi² = 6.30, df = 4 (P = 0.18); I² = 29%								
**1.1.2 Levocetirizine**								
✓ Elsekily 2024	8	6.02	20	9.5	5.52	10	15.1%	-1.50 [-5.82 , 2.82]
✕ Pandey 2019	10.44	3.86	50	6.35	4.3	50	0.0%	4.09 [2.49 , 5.69]
**Subtotal**			**20**			**10**	**15.1%**	**-1.50 [-5.82 , 2.82]**
Test for overall effect: Z = 0.68 (P = 0.50)								
Heterogeneity: Not applicable								
**Total (Wald[a])**			**160**			**131**	**100.0%**	**1.49 [-0.59 , 3.57]**

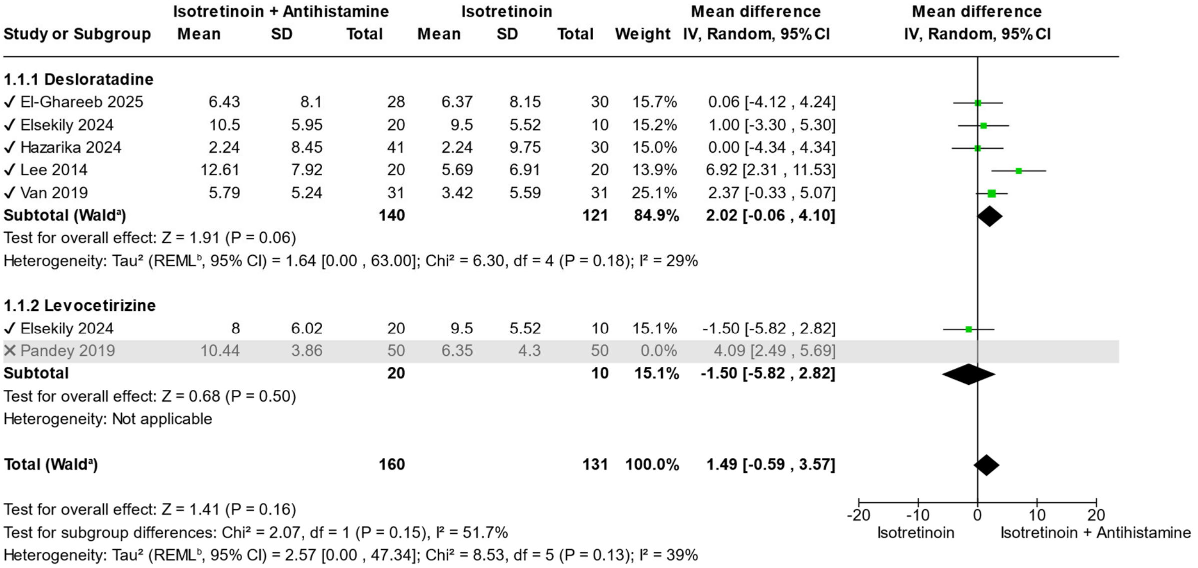

Test for overall effect: Z = 1.41 (P = 0.16)
Test for subgroup differences: Chi² = 2.07, df = 1 (P = 0.15), I² = 51.7%
Heterogeneity: Tau² (REML[b], 95% CI) = 2.57 [0.00 , 47.34]; Chi² = 8.53, df = 5 (P = 0.13); I² = 39%

Footnotes
[a]CI calculated by Wald-type method.
[b]Tau² calculated by Restricted Maximum-Likelihood method.

Figure S4. GAGS mean change from baseline till week 4

Study or Subgroup	Isotretinoin + Antihistamine Mean	SD	Total	Isotretinoin Mean	SD	Total	Weight	Mean difference IV, Random, 95% CI
**1.2.1 Desloratadine**								
Asilian 2015	7.93	6.85	28	8.17	7.96	28	15.1%	-0.24 [-4.13 , 3.65]
El-Ghareeb 2025	10.6	5.69	28	11.57	7.83	30	16.3%	-0.97 [-4.48 , 2.54]
Hazarika 2024	2.24	8.45	41	2.24	9.75	30	13.8%	0.00 [-4.34 , 4.34]
Lee 2014	18.41	7.32	20	10.49	7.05	20	13.5%	7.92 [3.47 , 12.37]
Van 2019	5.79	5.24	31	3.42	5.59	31	18.9%	2.37 [-0.33 , 5.07]
**Subtotal (Wald[a])**			**148**			**139**	**77.7%**	**1.71 [-1.22 , 4.65]**
Test for overall effect: Z = 1.15 (P = 0.25)								
Heterogeneity: Tau² (REML[b], 95% CI) = 7.49 [0.14 , >100]; Chi² = 11.49, df = 4 (P = 0.02); I² = 68%								
**1.2.2 Levocetirizine**								
Pandey 2019	10.44	3.86	50	6.35	4.3	50	22.3%	4.09 [2.49 , 5.69]
**Subtotal**			**50**			**50**	**22.3%**	**4.09 [2.49 , 5.69]**
Test for overall effect: Z = 5.00 (P < 0.00001)								
Heterogeneity: Not applicable								
**Total (Wald[a])**			**198**			**189**	**100.0%**	**2.24 [-0.19 , 4.66]**

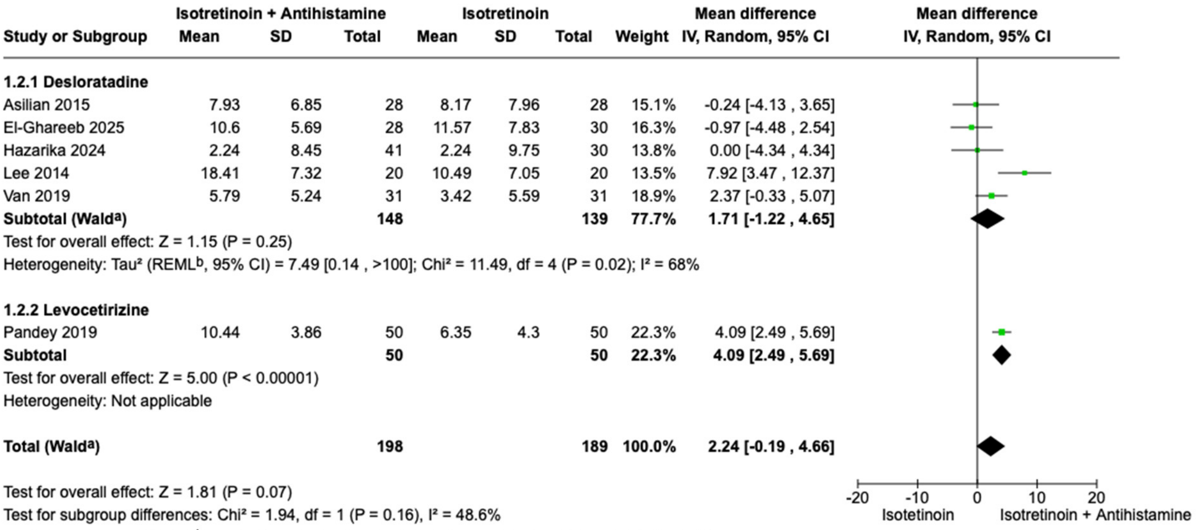

Test for overall effect: Z = 1.81 (P = 0.07)
Test for subgroup differences: Chi² = 1.94, df = 1 (P = 0.16), I² = 48.6%
Heterogeneity: Tau² (REML[b], 95% CI) = 6.20 [0.66 , 64.40]; Chi² = 16.04, df = 5 (P = 0.007); I² = 72%

Footnotes
[a]CI calculated by Wald-type method.
[b]Tau² calculated by Restricted Maximum-Likelihood method.

Figure S5. GAGS mean change from baseline till week 8

Study or Subgroup	Isotretinoin + Antihistamine Mean	SD	Total	Isotretinoin Mean	SD	Total	Weight	Mean difference IV, Random, 95% CI
**1.2.1 Desloratadine**								
✓ Asilian 2015	7.93	6.85	28	8.17	7.96	28	16.2%	-0.24 [-4.13 , 3.65]
✓ El-Ghareeb 2025	10.6	5.69	28	11.57	7.83	30	18.0%	-0.97 [-4.48 , 2.54]
✓ Hazarika 2024	2.24	8.45	41	2.24	9.75	30	14.3%	0.00 [-4.34 , 4.34]
✕ Lee 2014	18.41	7.32	20	10.49	7.05	20	0.0%	7.92 [3.47 , 12.37]
✓ Van 2019	5.79	5.24	31	3.42	5.59	31	22.4%	2.37 [-0.33 , 5.07]
**Subtotal (Wald[a])**			**128**			**119**	**70.8%**	**0.62 [-1.23 , 2.47]**
Test for overall effect: Z = 0.66 (P = 0.51)								
Heterogeneity: Tau² (REML[b], 95% CI) = 0.43 [0.00 , 26.74]; Chi² = 2.67, df = 3 (P = 0.45); I² = 12%								
**1.2.2 Levocetirizine**								
✓ Pandey 2019	10.44	3.86	50	6.35	4.3	50	29.2%	4.09 [2.49 , 5.69]
**Subtotal**			**50**			**50**	**29.2%**	**4.09 [2.49 , 5.69]**
Test for overall effect: Z = 5.00 (P < 0.00001)								
Heterogeneity: Not applicable								
**Total (Wald[a])**			**178**			**169**	**100.0%**	**1.51 [-0.62 , 3.64]**

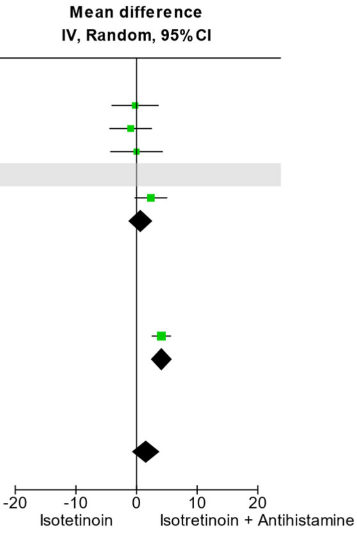

Test for overall effect: Z = 1.39 (P = 0.16)
Test for subgroup differences: Chi² = 7.72, df = 1 (P = 0.005), I² = 87.0%
Heterogeneity: Tau² (REML[b], 95% CI) = 3.38 [0.00 , 34.89]; Chi² = 10.74, df = 4 (P = 0.03); I² = 61%

Footnotes
[a]CI calculated by Wald-type method.
[b]Tau² calculated by Restricted Maximum-Likelihood method.

Figure S6. GAGS mean change from baseline till week 8

Study or Subgroup	Isotretinoin + Antihistamine Mean	SD	Total	Isotretinoin Mean	SD	Total	Weight	Mean difference IV, Random, 95% CI
**1.2.1 Desloratadine**								
✓ Asilian 2015	7.93	6.85	28	8.17	7.96	28	17.7%	-0.24 [-4.13 , 3.65]
✕ El-Ghareeb 2025	10.6	5.69	28	11.57	7.83	30	0.0%	-0.97 [-4.48 , 2.54]
✓ Hazarika 2024	2.24	8.45	41	2.24	9.75	30	16.0%	0.00 [-4.34 , 4.34]
✓ Lee 2014	18.41	7.32	20	10.49	7.05	20	15.6%	7.92 [3.47 , 12.37]
✓ Van 2019	5.79	5.24	31	3.42	5.59	31	22.9%	2.37 [-0.33 , 5.07]
**Subtotal (Wald[a])**			**120**			**109**	**72.2%**	**2.43 [-1.00 , 5.86]**
Test for overall effect: Z = 1.39 (P = 0.16)								
Heterogeneity: Tau² (REML[b], 95% CI) = 8.39 [0.00 , >100]; Chi² = 8.83, df = 3 (P = 0.03); I² = 70%								
**1.2.2 Levocetirizine**								
✓ Pandey 2019	10.44	3.86	50	6.35	4.3	50	27.8%	4.09 [2.49 , 5.69]
**Subtotal**			**50**			**50**	**27.8%**	**4.09 [2.49 , 5.69]**
Test for overall effect: Z = 5.00 (P < 0.00001)								
Heterogeneity: Not applicable								
**Total (Wald[a])**			**170**			**159**	**100.0%**	**2.87 [0.39 , 5.35]**

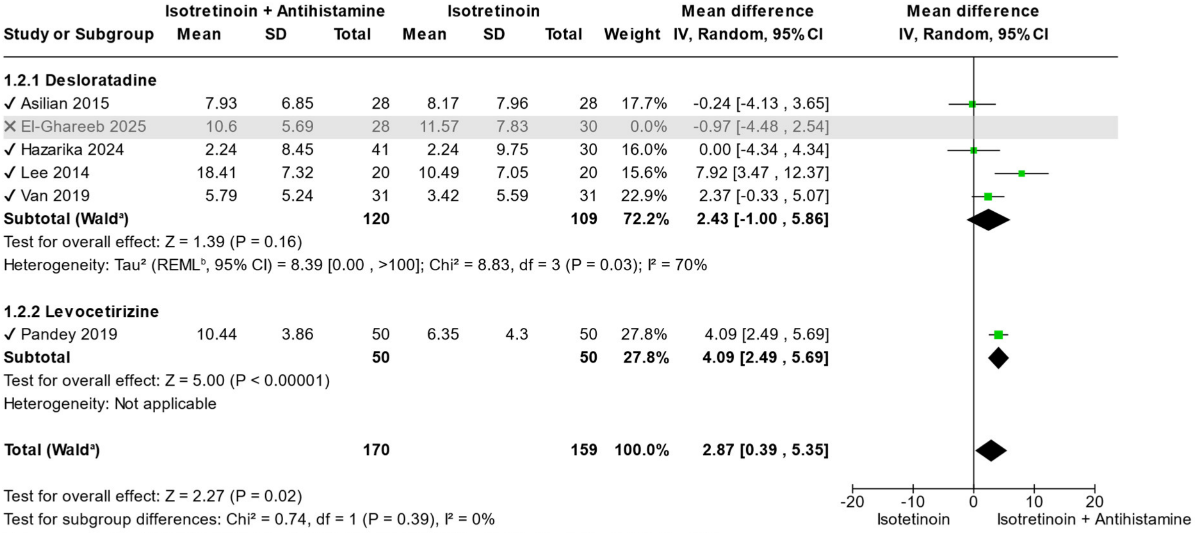

Test for overall effect: Z = 2.27 (P = 0.02)
Test for subgroup differences: Chi² = 0.74, df = 1 (P = 0.39), I² = 0%
Heterogeneity: Tau² (REML[b], 95% CI) = 5.10 [0.00 , 88.51]; Chi² = 10.92, df = 4 (P = 0.03); I² = 68%

Footnotes
[a]CI calculated by Wald-type method.
[b]Tau² calculated by Restricted Maximum-Likelihood method.

Figure S7. GAGS mean change from baseline till week 8

Study or Subgroup	Isotretinoin + Desloratadine Mean	SD	Total	Isotretinoin Mean	SD	Total	Weight	Mean difference IV, Random, 95% CI
Asilian 2015	13.43	5.04	28	13.51	5.71	28	20.4%	-0.08 [-2.90 , 2.74]
Mansoor 2022	22.13	3.48	54	19.76	4.84	54	54.9%	2.37 [0.78 , 3.96]
Van 2019	19.19	4.92	31	16.25	5.3	31	24.6%	2.94 [0.39 , 5.49]
**Total (Wald[a])**			**113**			**113**	**100.0%**	**2.01 [0.68 , 3.34]**

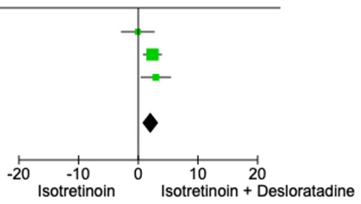

Test for overall effect: Z = 2.96 (P = 0.003)
Test for subgroup differences: Not applicable
Heterogeneity: Tau² (REML[b]) = 0.18; Chi² = 2.82, df = 2 (P = 0.24); I² = 12%

Footnotes
[a]CI calculated by Wald-type method.
[b]Tau² calculated by Restricted Maximum-Likelihood method.

Figure S8. GAGS mean change from baseline till week 16

Study or Subgroup	Isotretinoin + Desloratadine Mean	SD	Total	Isotretinoin Mean	SD	Total	Weight	Mean difference IV, Random, 95% CI
✓ Asilian 2015	13.43	5.04	28	13.51	5.71	28	47.9%	-0.08 [-2.90 , 2.74]
✕ Mansoor 2022	22.13	3.48	54	19.76	4.84	54	0.0%	2.37 [0.78 , 3.96]
✓ Van 2019	19.19	4.92	31	16.25	5.3	31	52.1%	2.94 [0.39 , 5.49]
**Total (Wald[a])**			**59**			**59**	**100.0%**	**1.49 [-1.46 , 4.45]**

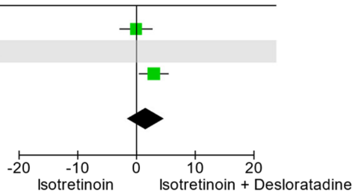

Test for overall effect: Z = 0.99 (P = 0.32)

Heterogeneity: Tau² (REML[b]) = 2.68; Chi² = 2.43, df = 1 (P = 0.12); I² = 59%

Footnotes
[a]CI calculated by Wald-type method.
[b]Tau² calculated by Restricted Maximum-Likelihood method.

Figure S9. GAGS mean change from baseline till week 16

Study or Subgroup	Isotretinoin + Desloratadine Mean	SD	Total	Isotretinoin Mean	SD	Total	Weight	Mean difference IV, Random, 95% CI
✓ Asilian 2015	13.43	5.04	28	13.51	5.71	28	38.2%	-0.08 [-2.90 , 2.74]
✓ Mansoor 2022	22.13	3.48	54	19.76	4.84	54	61.8%	2.37 [0.78 , 3.96]
✕ Van 2019	19.19	4.92	31	16.25	5.3	31	0.0%	2.94 [0.39 , 5.49]
**Total (Wald[a])**			**82**			**82**	**100.0%**	**1.43 [-0.90 , 3.77]**

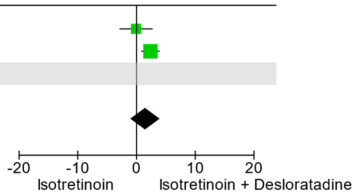

Test for overall effect: Z = 1.20 (P = 0.23)

Heterogeneity: Tau² (REML[b]) = 1.64; Chi² = 2.20, df = 1 (P = 0.14); I² = 55%

Footnotes
[a]CI calculated by Wald-type method.
[b]Tau² calculated by Restricted Maximum-Likelihood method.

Figure S10. GAGS mean change from baseline till week 16

Study or Subgroup	Isotretinoin + Antihistamine Mean	SD	Total	Isotretinoin Mean	SD	Total	Weight	Mean difference IV, Random, 95% CI
**1.18.1 Desloratadine**								
Asilian 2015	20.19	4.69	30	17.93	5.37	30	52.6%	2.26 [-0.29 , 4.81]
Elsekily 2024	31	5.97	20	28	4.9	10	21.3%	3.00 [-1.01 , 7.01]
**Subtotal (Wald[a])**			**50**			**40**	**73.9%**	**2.47 [0.32 , 4.63]**
Test for overall effect: Z = 2.25 (P = 0.02)								
Heterogeneity: Tau² (REML[b], 95% CI) = 0.00 [0.00 , >100]; Chi² = 0.09, df = 1 (P = 0.76); I² = 0%								
**1.18.2 Levocetirizine**								
Elsekily 2024	31	4.51	20	28	4.9	10	26.1%	3.00 [-0.62 , 6.62]
**Subtotal**			**20**			**10**	**26.1%**	**3.00 [-0.62 , 6.62]**
Test for overall effect: Z = 1.62 (P = 0.10)								
Heterogeneity: Not applicable								
**Total (Wald[a])**			**70**			**50**	**100.0%**	**2.61 [0.76 , 4.46]**
95% prediction interval								[0.76 , 4.46]

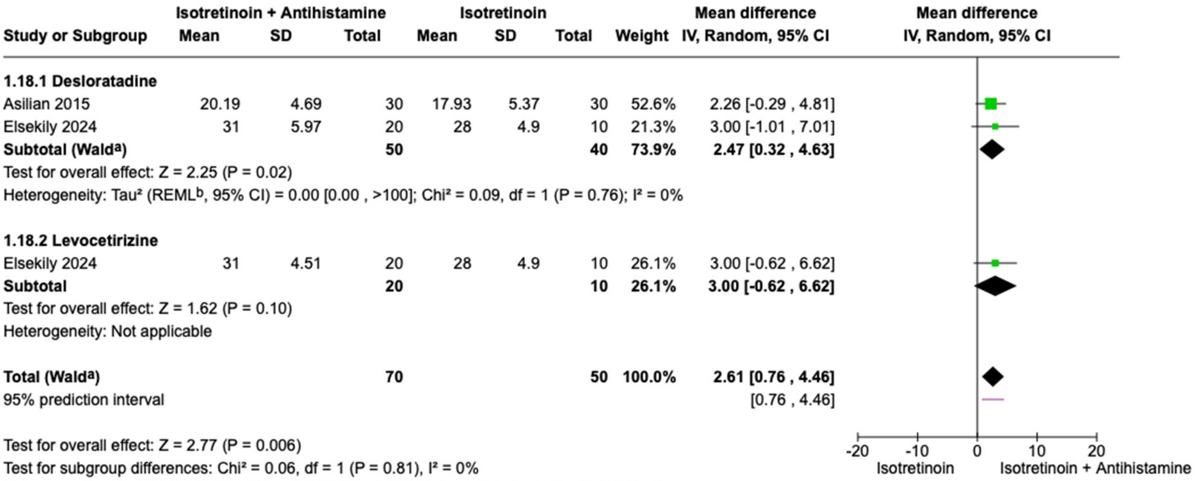

Test for overall effect: Z = 2.77 (P = 0.006)
Test for subgroup differences: Chi² = 0.06, df = 1 (P = 0.81), I² = 0%
Heterogeneity: Tau² (REML[b], 95% CI) = 0.00 [0.00 , 4.82]; Chi² = 0.15, df = 2 (P = 0.93); I² = 0%

Footnotes
[a]CI calculated by Wald-type method.
[b]Tau² calculated by Restricted Maximum-Likelihood method.

Figure S11. GAGS mean change from baseline till week 24

Study or Subgroup	Isotretinoin + Antihistamine Events	Total	Isotretinoin Events	Total	Weight	Odds ratio IV, Random, 95% CI
**1.1.1 Desloratadine**						
✓ Elsekily 2024	3	20	3	10	13.1%	0.41 [0.07 , 2.56]
✓ Hazarika 2024	4	41	5	30	22.0%	0.54 [0.13 , 2.21]
✓ Lee 2014	1	20	6	20	8.8%	0.12 [0.01 , 1.14]
✓ Van 2019	7	31	14	31	36.2%	0.35 [0.12 , 1.06]
**Subtotal (Wald[a])**		**112**		**91**	**80.2%**	**0.36 [0.17 , 0.76]**
Total events:	15		28			
Test for overall effect: Z = 2.69 (P = 0.007)						
Heterogeneity: Tau² (REML[b]) = 0.00; Chi² = 1.24, df = 3 (P = 0.74); I² = 0%						
**1.1.2 Levocetirizine**						
✓ Elsekily 2024	2	20	2	10	9.7%	0.44 [0.05 , 3.74]
✓ Pandey 2019	1	50	13	50	10.1%	0.06 [0.01 , 0.46]
✕ Yosef 2017	6	25	2	25	0.0%	3.63 [0.66 , 20.11]
**Subtotal (Wald[a])**		**70**		**60**	**19.8%**	**0.16 [0.02 , 1.16]**
Total events:	3		15			
Test for overall effect: Z = 1.81 (P = 0.07)						
Heterogeneity: Tau² (REML[b]) = 0.92; Chi² = 1.80, df = 1 (P = 0.18); I² = 44%						
**Total (Wald[a])**		**182**		**151**	**100.0%**	**0.31 [0.16 , 0.60]**
Total events:	18		43			

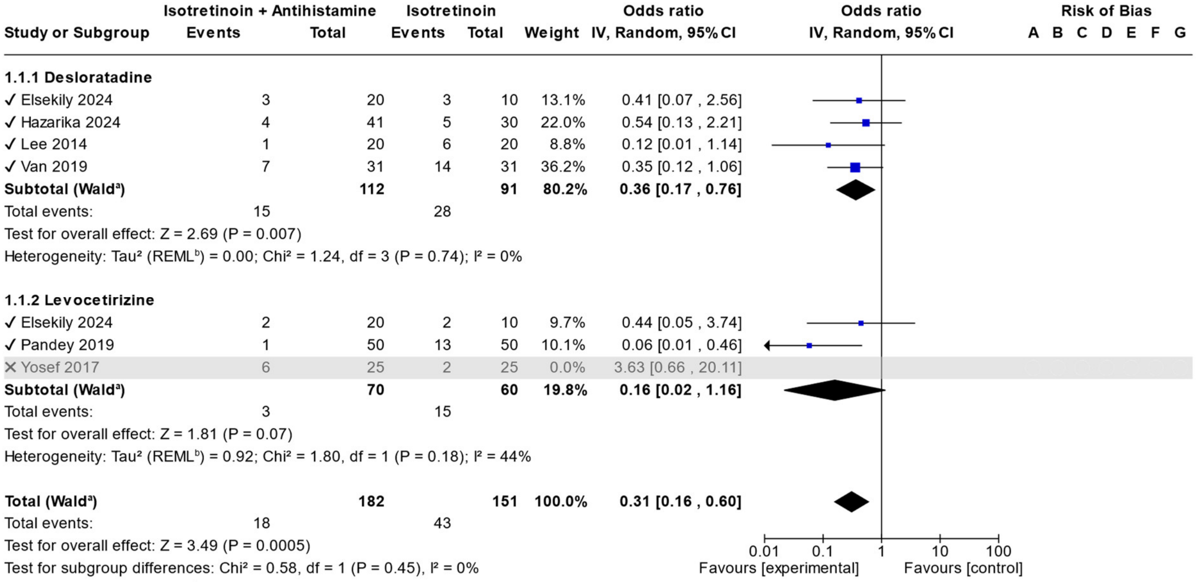

Test for overall effect: Z = 3.49 (P = 0.0005)
Test for subgroup differences: Chi² = 0.58, df = 1 (P = 0.45), I² = 0%
Heterogeneity: Tau² (REML[b]) = 0.00; Chi² = 4.01, df = 5 (P = 0.55); I² = 0%

Footnotes
[a]CI calculated by Wald-type method.
[b]Tau² calculated by Restricted Maximum-Likelihood method.

Risk of bias legend
(A) Random sequence generation (selection bias)
(B) Allocation concealment (selection bias)
(C) Blinding of participants and personnel (performance bias)
(D) Blinding of outcome assessment (detection bias)
(E) Incomplete outcome data (attrition bias)
(F) Selective reporting (reporting bias)
(G) Other bias

Figure S12. Acne flare-ups side effect

Study or Subgroup	Isotretinoin + Anthistamine Events	Isotretinoin + Anthistamine Total	Isotretinoin Events	Isotretinoin Total	Weight	Odds ratio IV, Random, 95% CI
**1.7.1 Desloratadine**						
✕ Dhaher 2018	22	38	34	38	0.0%	0.16 [0.05 , 0.55]
✓ Elsekily 2024	19	20	9	10	5.2%	2.11 [0.12 , 37.72]
✓ Hazarika 2024	18	41	18	30	47.5%	0.52 [0.20 , 1.36]
✓ Lee 2014	15	20	18	20	13.7%	0.33 [0.06 , 1.97]
**Subtotal (Wald[a])**		**81**		**60**	**66.4%**	**0.53 [0.24 , 1.19]**
Total events:	52		45			
Test for overall effect: Z = 1.54 (P = 0.12)						
Heterogeneity: Tau² (REML[b], 95% CI) = 0.00 [0.00 , 34.98]; Chi² = 1.15, df = 2 (P = 0.56); I² = 0%						
**1.7.2 Levocetirizine**						
✓ Elsekily 2024	17	20	9	10	7.5%	0.63 [0.06 , 6.96]
✓ Yosef 2017	17	25	20	25	26.0%	0.53 [0.15 , 1.93]
**Subtotal (Wald[a])**		**45**		**35**	**33.6%**	**0.55 [0.18 , 1.72]**
Total events:	34		29			
Test for overall effect: Z = 1.02 (P = 0.31)						
Heterogeneity: Tau² (REML[b], 95% CI) = 0.00 [0.00 , 13.73]; Chi² = 0.01, df = 1 (P = 0.90); I² = 0%						
**Total (Wald[a])**		**126**		**95**	**100.0%**	**0.54 [0.28 , 1.04]**
95% prediction interval						[0.28 , 1.04]
Total events:	86		74			
Test for overall effect: Z = 1.85 (P = 0.06)						
Test for subgroup differences: Chi² = 0.00, df = 1 (P = 0.96), I² = 0%						
Heterogeneity: Tau² (REML[b], 95% CI) = 0.00 [0.00 , 2.25]; Chi² = 1.16, df = 4 (P = 0.88); I² = 0%						

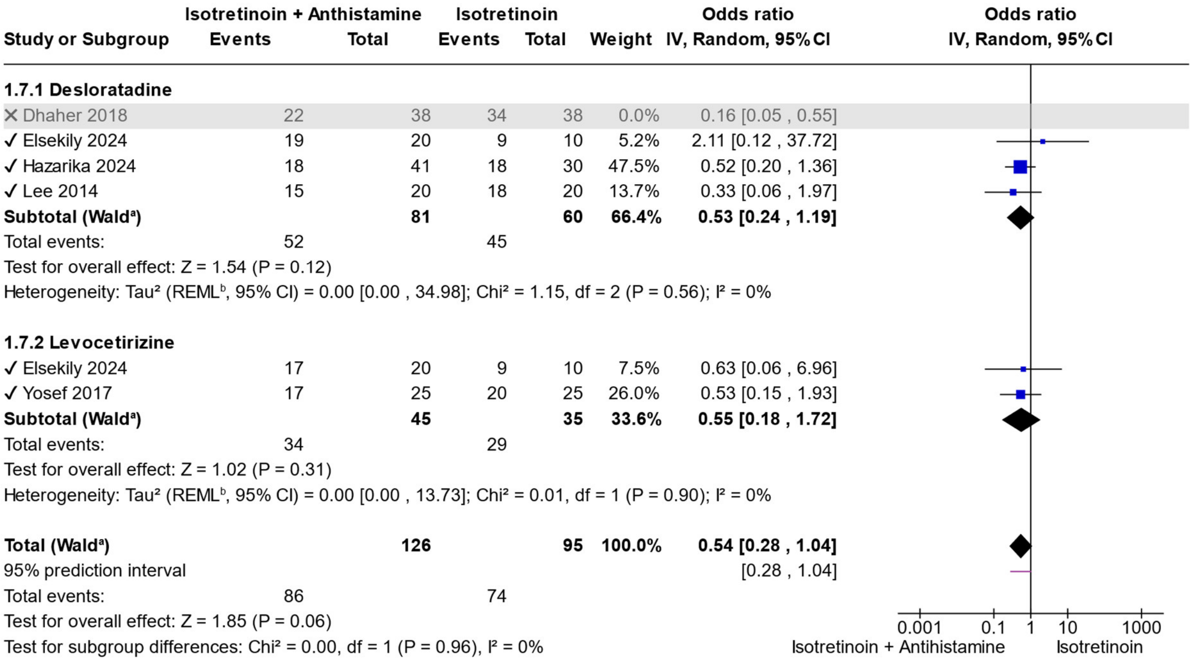

Footnotes

[a]CI calculated by Wald-type method.

[b]Tau² calculated by Restricted Maximum-Likelihood method.

Figure S13. Cheilitis side effect

Study or Subgroup	Isotretinoin + Anthistamine Events	Isotretinoin + Anthistamine Total	Isotretinoin Events	Isotretinoin Total	Weight	Odds ratio IV, Random, 95% CI
**1.8.1 Desloratadine**						
✓ Elsekily 2024	2	20	7	10	11.5%	0.05 [0.01 , 0.35]
✓ Hazarika 2024	4	41	10	30	27.9%	0.22 [0.06 , 0.78]
✓ Lee 2014	3	20	9	20	20.0%	0.22 [0.05 , 0.98]
✓ Van 2019	4	31	20	31	27.8%	0.08 [0.02 , 0.29]
**Subtotal (Wald[a])**		**112**		**91**	**87.3%**	**0.13 [0.06 , 0.27]**
Total events:	13		46			
Test for overall effect: Z = 5.53 (P < 0.00001)						
Heterogeneity: Tau² (REML[b], 95% CI) = 0.00 [0.00 , 7.09]; Chi² = 2.52, df = 3 (P = 0.47); I² = 0%						
**1.8.2 Levocetirizine**						
✓ Elsekily 2024	4	20	8	10	12.7%	0.06 [0.01 , 0.42]
✕ Yosef 2017	7	25	7	25	0.0%	1.00 [0.29 , 3.44]
**Subtotal**		**20**		**10**	**12.7%**	**0.06 [0.01 , 0.42]**
Total events:	4		8			
Test for overall effect: Z = 2.86 (P = 0.004)						
Heterogeneity: Not applicable						
**Total (Wald[a])**		**132**		**101**	**100.0%**	**0.12 [0.06 , 0.23]**
95% prediction interval						[0.06 , 0.23]
Total events:	17		54			
Test for overall effect: Z = 6.19 (P < 0.00001)						
Test for subgroup differences: Chi² = 0.50, df = 1 (P = 0.48), I² = 0%						
Heterogeneity: Tau² (REML[b], 95% CI) = 0.00 [0.00 , 3.41]; Chi² = 3.02, df = 4 (P = 0.55); I² = 0%						

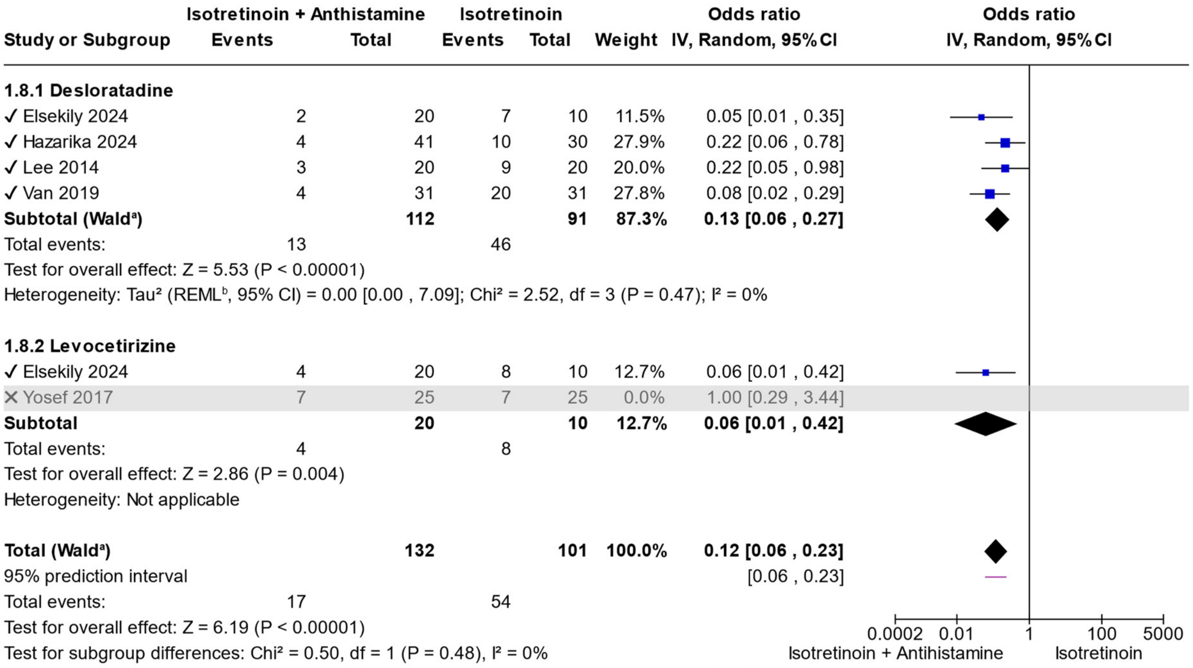

Footnotes

[a]CI calculated by Wald-type method.

[b]Tau² calculated by Restricted Maximum-Likelihood method.

Figure S14. Pruritus side effect

Study or Subgroup	Isotretinoin + Antihistamine Mean	SD	Total	Isotretinoin Mean	SD	Total	Weight	Mean difference IV, Random, 95% CI
**1.14.1 Desloratadine**								
Elsekily 2024	12	12.14	20	5.7	11.76	10	14.8%	6.30 [-2.72 , 15.32]
Hazarika 2024	2	2.96	41	2	2.52	30	41.0%	0.00 [-1.28 , 1.28]
Lee 2014	16.54	14.32	20	7.17	15.57	20	14.2%	9.37 [0.10 , 18.64]
**Subtotal (Wald[a])**			**81**			**60**	**70.0%**	**3.77 [-2.32 , 9.87]**
Test for overall effect: Z = 1.21 (P = 0.23)								
Heterogeneity: Tau² (REML[b], 95% CI) = 18.30 [0.00 , >182.97370308376557]; Chi² = 5.59, df = 2 (P = 0.06); I² = 62%								
**1.14.2 Levocetirizine**								
Elsekily 2024	7	11.48	20	5.7	11.76	10	15.1%	1.30 [-7.56 , 10.16]
Pandey 2019	21.77	24.16	50	13.9	21.58	50	14.8%	7.87 [-1.11 , 16.85]
**Subtotal (Wald[a])**			**70**			**60**	**30.0%**	**4.54 [-1.90 , 10.98]**
Test for overall effect: Z = 1.38 (P = 0.17)								
Heterogeneity: Tau² (REML[b], 95% CI) = 0.88 [0.00 , >100]; Chi² = 1.04, df = 1 (P = 0.31); I² = 4%								
**Total (Wald[a])**			**151**			**120**	**100.0%**	**3.63 [-0.66 , 7.92]**
95% prediction interval								[-4.21 , 11.47]

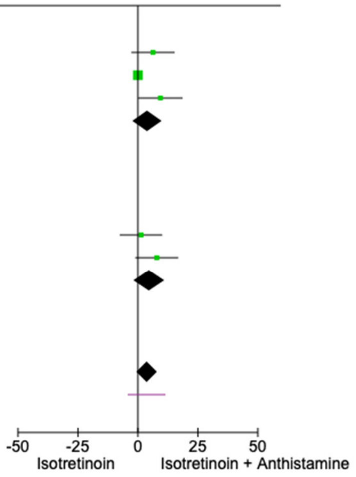

Test for overall effect: Z = 1.66 (P = 0.10)
Test for subgroup differences: Chi² = 0.03, df = 1 (P = 0.87), I² = 0%
Heterogeneity: Tau² (REML[b], 95% CI) = 11.23 [0.00 , >112.30003350156275]; Chi² = 8.31, df = 4 (P = 0.08); I² = 50%

Footnotes
[a]CI calculated by Wald-type method.
[b]Tau² calculated by Restricted Maximum-Likelihood method.

Figure S15. Inflammatory lesions count mean change from baseline till week 4

Study or Subgroup	Isotretinoin + Antihistamine Mean	SD	Total	Isotretinoin Mean	SD	Total	Weight	Mean difference IV, Random, 95% CI
**1.14.1 Desloratadine**								
✓ Elsekily 2024	12	12.14	20	5.7	11.76	10	25.0%	6.30 [-2.72 , 15.32]
✕ Hazarika 2024	2	2.96	41	2	2.52	30	0.0%	0.00 [-1.28 , 1.28]
✓ Lee 2014	16.54	14.32	20	7.17	15.57	20	23.7%	9.37 [0.10 , 18.64]
**Subtotal (Wald[a])**			**40**			**30**	**48.7%**	**7.79 [1.33 , 14.26]**
Test for overall effect: Z = 2.36 (P = 0.02)								
Heterogeneity: Tau² (REML[b], 95% CI) = 0.00 [0.00 , >100]; Chi² = 0.22, df = 1 (P = 0.64); I² = 0%								
**1.14.2 Levocetirizine**								
✓ Elsekily 2024	7	11.48	20	5.7	11.76	10	26.0%	1.30 [-7.56 , 10.16]
✓ Pandey 2019	21.77	24.16	50	13.9	21.58	50	25.3%	7.87 [-1.11 , 16.85]
**Subtotal (Wald[a])**			**70**			**60**	**51.3%**	**4.54 [-1.90 , 10.98]**
Test for overall effect: Z = 1.38 (P = 0.17)								
Heterogeneity: Tau² (REML[b], 95% CI) = 0.88 [0.00 , >100]; Chi² = 1.04, df = 1 (P = 0.31); I² = 4%								
**Total (Wald[a])**			**110**			**90**	**100.0%**	**6.13 [1.61 , 10.64]**
95% prediction interval								[1.61 , 10.64]

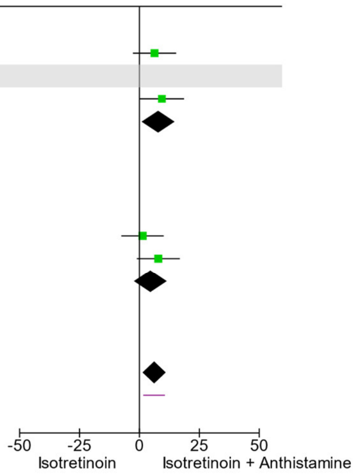

Test for overall effect: Z = 2.66 (P = 0.008)
Test for subgroup differences: Chi² = 0.49, df = 1 (P = 0.48), I² = 0%
Heterogeneity: Tau² (REML[b], 95% CI) = 0.00 [0.00 , >100]; Chi² = 1.76, df = 3 (P = 0.62); I² = 0%

Footnotes
[a]CI calculated by Wald-type method.
[b]Tau² calculated by Restricted Maximum-Likelihood method.

Figure S16. Inflammatory lesions count mean change from baseline till week 4

Study or Subgroup	Isotretinoin + Anthistamine Mean	SD	Total	Isotretinoin Mean	SD	Total	Weight	Mean difference IV, Random, 95% CI
**1.13.1 Desloratadine**								
Asilian 2015	14.34	11.73	28	10.69	11.44	28	25.7%	3.65 [-2.42 , 9.72]
Hazarika 2024	3	2.65	41	4.5	2.78	30	35.2%	-1.50 [-2.78 , -0.22]
Lee 2014	21.89	14.35	20	13.61	15.6	20	18.6%	8.28 [-1.01 , 17.57]
**Subtotal (Wald[a])**			**89**			**78**	**79.5%**	**2.10 [-3.38 , 7.57]**
Test for overall effect: Z = 0.75 (P = 0.45)								
Heterogeneity: Tau^2 (REML[b], 95% CI) = 15.63 [0.00 , >156.34684461428867]; Chi^2 = 6.64, df = 2 (P = 0.04); I^2 = 69%								
**1.13.2 Levocetirizin**								
Pandey 2019	29.05	22.33	50	18.37	20.08	50	20.5%	10.68 [2.36 , 19.00]
**Subtotal**			**50**			**50**	**20.5%**	**10.68 [2.36 , 19.00]**
Test for overall effect: Z = 2.51 (P = 0.01)								
Heterogeneity: Not applicable								
**Total (Wald[a])**			**139**			**128**	**100.0%**	**4.14 [-1.63 , 9.91]**
95% prediction interval								[-7.09 , 15.37]

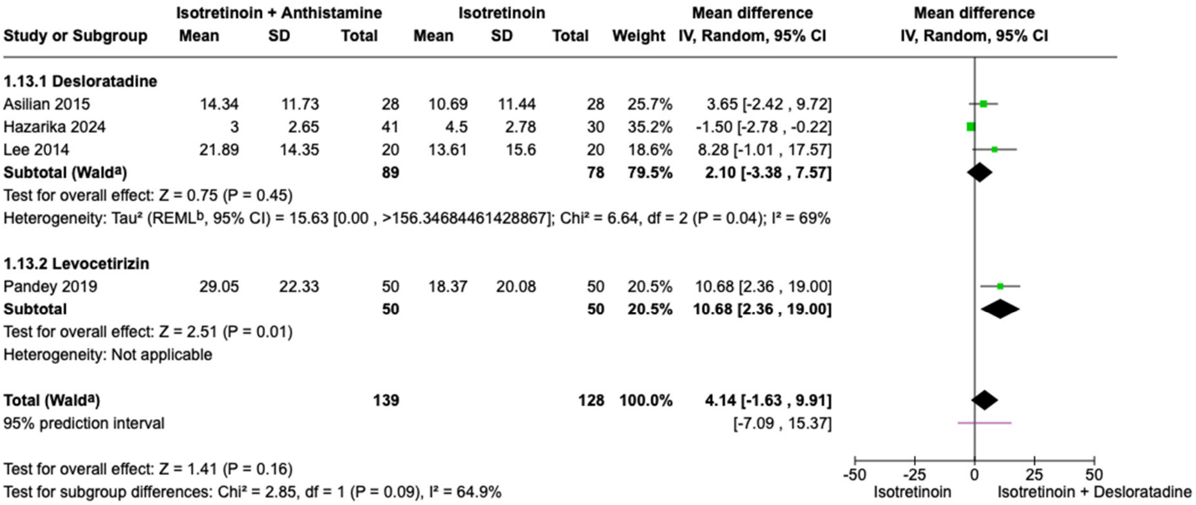

Test for overall effect: Z = 1.41 (P = 0.16)
Test for subgroup differences: Chi^2 = 2.85, df = 1 (P = 0.09), I^2 = 64.9%
Heterogeneity: Tau^2 (REML[b], 95% CI) = 24.18 [2.52 , >241.7849514015066]; Chi^2 = 14.18, df = 3 (P = 0.003); I^2 = 76%

Footnotes
[a]CI calculated by Wald-type method.
[b]Tau^2 calculated by Restricted Maximum-Likelihood method.

Figure S17. Inflammatory lesions count mean change from baseline till week 8

Study or Subgroup	Isotretinoin + Anthistamine Mean	SD	Total	Isotretinoin Mean	SD	Total	Weight	Mean difference IV, Random, 95% CI
**1.13.1 Desloratadine**								
✓ Asilian 2015	14.34	11.73	28	10.69	11.44	28	48.6%	3.65 [-2.42 , 9.72]
✕ Hazarika 2024	3	2.65	41	4.5	2.78	30	0.0%	-1.50 [-2.78 , -0.22]
✓ Lee 2014	21.89	14.35	20	13.61	15.6	20	23.2%	8.28 [-1.01 , 17.57]
**Subtotal (Wald[a])**			**48**			**48**	**71.7%**	**5.04 [-0.05 , 10.12]**
Test for overall effect: Z = 1.94 (P = 0.05)								
Heterogeneity: Tau^2 (REML[b], 95% CI) = 0.00 [0.00 , >100]; Chi^2 = 0.67, df = 1 (P = 0.41); I^2 = 0%								
**1.13.2 Levocetirizin**								
✓ Pandey 2019	29.05	22.33	50	18.37	20.08	50	28.3%	10.68 [2.36 , 19.00]
**Subtotal**			**50**			**50**	**28.3%**	**10.68 [2.36 , 19.00]**
Test for overall effect: Z = 2.51 (P = 0.01)								
Heterogeneity: Not applicable								
**Total (Wald[a])**			**98**			**98**	**100.0%**	**6.71 [2.03 , 11.39]**
95% prediction interval								[1.21 , 12.21]

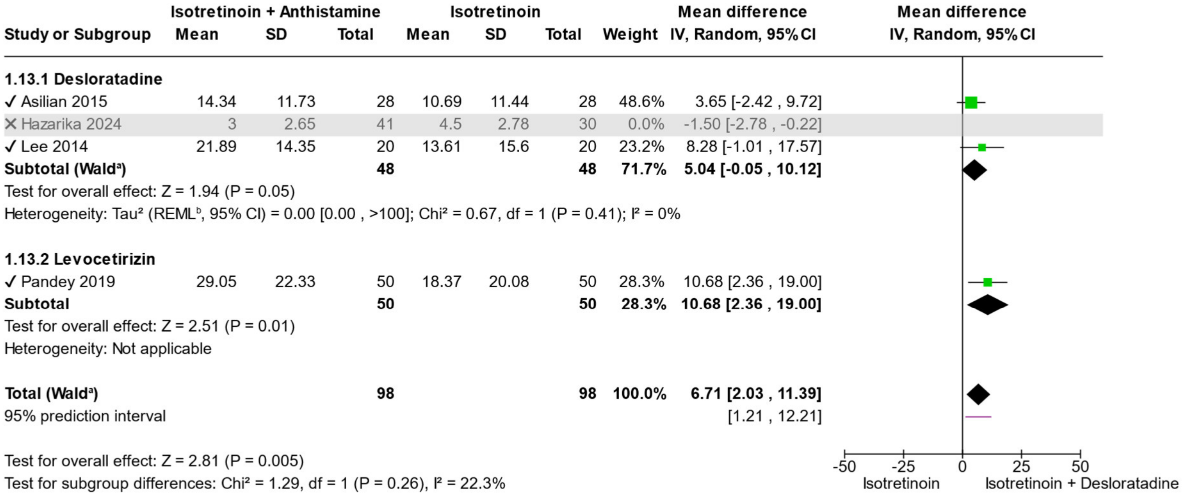

Test for overall effect: Z = 2.81 (P = 0.005)
Test for subgroup differences: Chi^2 = 1.29, df = 1 (P = 0.26), I^2 = 22.3%
Heterogeneity: Tau^2 (REML[b], 95% CI) = 2.16 [0.00 , >100]; Chi^2 = 1.96, df = 2 (P = 0.38); I^2 = 12%

Footnotes
[a]CI calculated by Wald-type method.
[b]Tau^2 calculated by Restricted Maximum-Likelihood method.

Figure S18. Inflammatory lesions count mean change from baseline till week 8

	Isotretinoin + Anthistamine			Isotretinoin				Mean difference
Study or Subgroup	Mean	SD	Total	Mean	SD	Total	Weight	IV, Random, 95% CI
**1.12.1 Desloratadine**								
✓ Dhaher 2018	49.6	17	38	36.6	15.7	38	30.9%	13.00 [5.64 , 20.36]
✓ Elsekily 2024	33.4	13.29	20	30.5	14.64	10	16.0%	2.90 [-7.88 , 13.68]
✕ Hazarika 2024	6	2.96	41	6.5	3.03	30	0.0%	-0.50 [-1.91 , 0.91]
✓ Lee 2014	23.04	14.42	20	13.95	15.18	20	21.3%	9.09 [-0.09 , 18.27]
**Subtotal (Wald[a])**			**78**			**68**	**68.1%**	**9.42 [3.96 , 14.88]**
Test for overall effect: Z = 3.38 (P = 0.0007)								
Heterogeneity: Tau² (REML[b], 95% CI) = 2.96 [0.00 , >100]; Chi² = 2.32, df = 2 (P = 0.31); I² = 12%								
**1.12.2 Levocetirizine**								
✓ Elsekily 2024	31.9	13.7	20	30.5	14.64	10	15.7%	1.40 [-9.48 , 12.28]
✓ Pandey 2019	32.69	29.16	50	24.45	25.48	50	16.1%	8.24 [-2.49 , 18.97]
**Subtotal (Wald[a])**			**70**			**60**	**31.9%**	**4.87 [-2.77 , 12.51]**
Test for overall effect: Z = 1.25 (P = 0.21)								
Heterogeneity: Tau² (REML[b], 95% CI) = 0.00 [0.00 , >100]; Chi² = 0.77, df = 1 (P = 0.38); I² = 0%								
**Total (Wald[a])**			**148**			**128**	**100.0%**	**7.96 [3.42 , 12.50]**
95% prediction interval								[2.20 , 13.72]

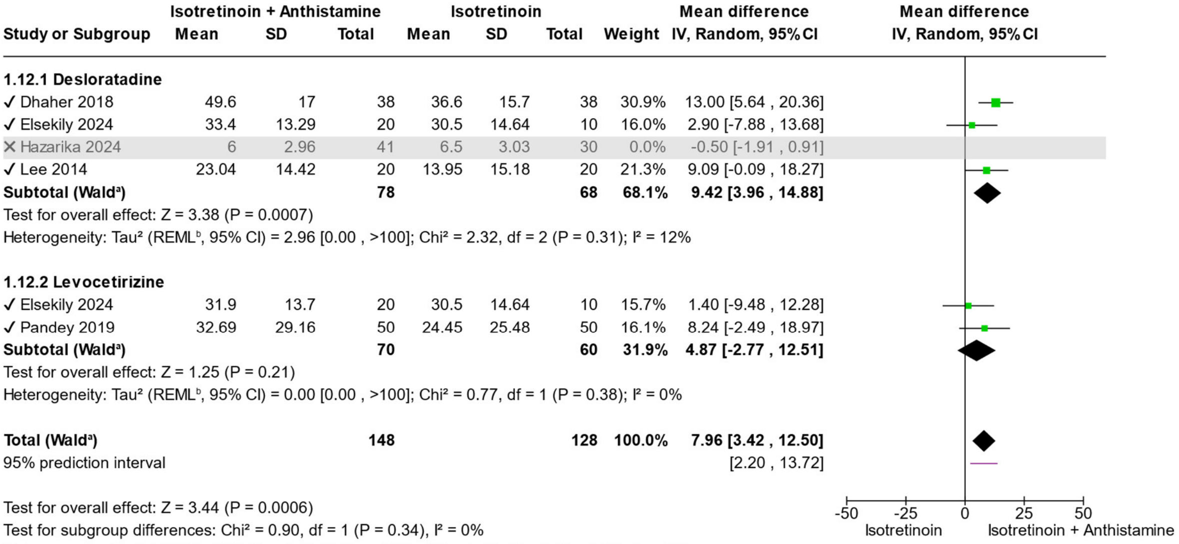

Test for overall effect: Z = 3.44 (P = 0.0006)
Test for subgroup differences: Chi² = 0.90, df = 1 (P = 0.34), I² = 0%
Heterogeneity: Tau² (REML[b], 95% CI) = 3.28 [0.00 , >100]; Chi² = 4.10, df = 4 (P = 0.39); I² = 12%

Footnotes
[a]CI calculated by Wald-type method.
[b]Tau² calculated by Restricted Maximum-Likelihood method.

Figure S19. Inflammatory lesions count mean change from baseline till week 12

	Isotretinoin + Antihistamine			Isotretinoin				Mean difference
Study or Subgroup	Mean	SD	Total	Mean	SD	Total	Weight	IV, Random, 95% CI
**1.15.1 Desloratadine**								
Dhaher 2018	4	3	38	2	3.6	38	40.2%	2.00 [0.51 , 3.49]
Elsekily 2024	7.2	12.8	20	2.8	13.6	10	9.9%	4.40 [-5.73 , 14.53]
Hazarika 2024	1	8.69	41	2	7.05	30	30.0%	-1.00 [-4.67 , 2.67]
Lee 2014	24.34	17.27	20	11.85	18.92	20	8.4%	12.49 [1.26 , 23.72]
**Subtotal (Wald[a])**			**119**			**98**	**88.5%**	**1.84 [-0.99 , 4.66]**
Test for overall effect: Z = 1.27 (P = 0.20)								
Heterogeneity: Tau² (REML[b], 95% CI) = 3.15 [0.00 , >100]; Chi² = 6.05, df = 3 (P = 0.11); I² = 39%								
**1.15.2 Levocetirizine**								
Elsekily 2024	7.4	12.21	20	2.8	13.6	10	10.1%	4.60 [-5.38 , 14.58]
Pandey 2019	69.18	93.26	50	31.26	60.77	50	1.3%	37.92 [7.07 , 68.77]
**Subtotal (Wald[a])**			**70**			**60**	**11.5%**	**17.93 [-14.06 , 49.93]**
Test for overall effect: Z = 1.10 (P = 0.27)								
Heterogeneity: Tau² (REML[b], 95% CI) = 418.23 [0.00 , >4182.318925000003]; Chi² = 4.06, df = 1 (P = 0.04); I² = 75%								
**Total (Wald[a])**			**189**			**158**	**100.0%**	**2.97 [-0.66 , 6.60]**
95% prediction interval								[-3.65 , 9.59]

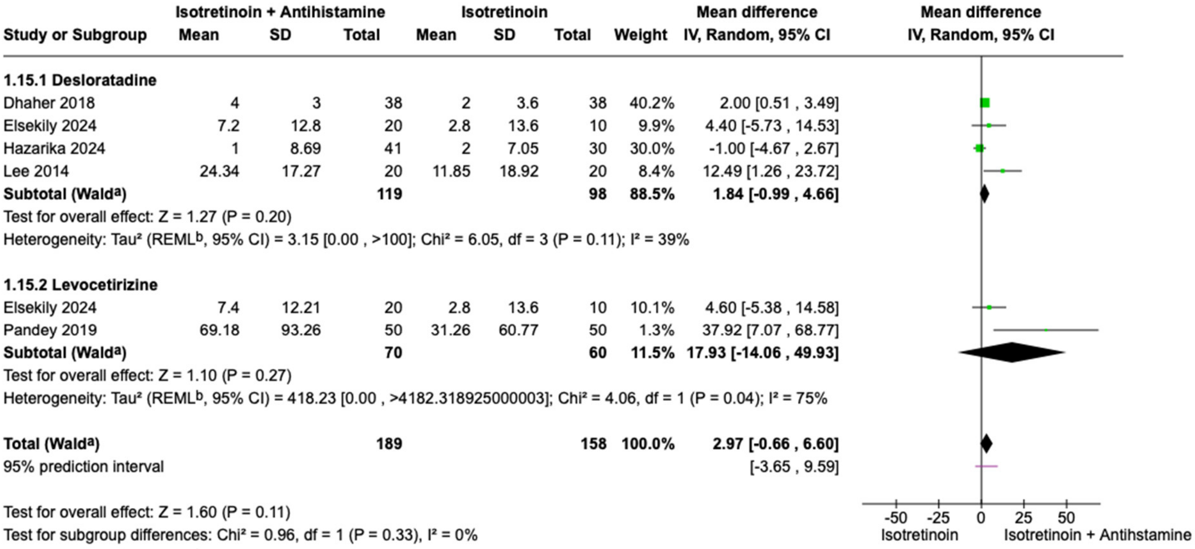

Test for overall effect: Z = 1.60 (P = 0.11)
Test for subgroup differences: Chi² = 0.96, df = 1 (P = 0.33), I² = 0%
Heterogeneity: Tau² (REML[b], 95% CI) = 7.97 [0.00 , >100]; Chi² = 11.59, df = 5 (P = 0.04); I² = 52%

Footnotes
[a]CI calculated by Wald-type method.
[b]Tau² calculated by Restricted Maximum-Likelihood method.

Figure S20. Non-inflammatory lesions count mean change from baseline till week 4

Study or Subgroup	Isotretinoin + Antihistamine Mean	SD	Total	Isotretinoin Mean	SD	Total	Weight	Mean difference IV, Random, 95% CI
**1.15.1 Desloratadine**								
✓ Dhaher 2018	4	3	38	2	3.6	38	49.9%	2.00 [0.51 , 3.49]
✓ Elsekily 2024	7.2	12.8	20	2.8	13.6	10	16.5%	4.40 [-5.73 , 14.53]
✕ Hazarika 2024	1	8.69	41	2	7.05	30	0.0%	-1.00 [-4.67 , 2.67]
✓ Lee 2014	24.34	17.27	20	11.85	18.92	20	14.3%	12.49 [1.26 , 23.72]
**Subtotal (Wald[a])**			**78**			**68**	**80.7%**	**4.26 [-1.11 , 9.64]**
Test for overall effect: Z = 1.55 (P = 0.12)								
Heterogeneity: Tau² (REML[b], 95% CI) = 11.34 [0.00 , >113.42648461556084]; Chi² = 3.48, df = 2 (P = 0.18); I² = 43%								
**1.15.2 Levocetirizine**								
✓ Elsekily 2024	7.4	12.21	20	2.8	13.6	10	16.8%	4.60 [-5.38 , 14.58]
✓ Pandey 2019	69.18	93.26	50	31.26	60.77	50	2.5%	37.92 [7.07 , 68.77]
**Subtotal (Wald[a])**			**70**			**60**	**19.3%**	**17.93 [-14.06 , 49.93]**
Test for overall effect: Z = 1.10 (P = 0.27)								
Heterogeneity: Tau² (REML[b], 95% CI) = 418.23 [0.00 , >4182.318925000003]; Chi² = 4.06, df = 1 (P = 0.04); I² = 75%								
**Total (Wald[a])**			**148**			**128**	**100.0%**	**5.22 [0.25 , 10.20]**
95% prediction interval								[-3.27 , 13.71]

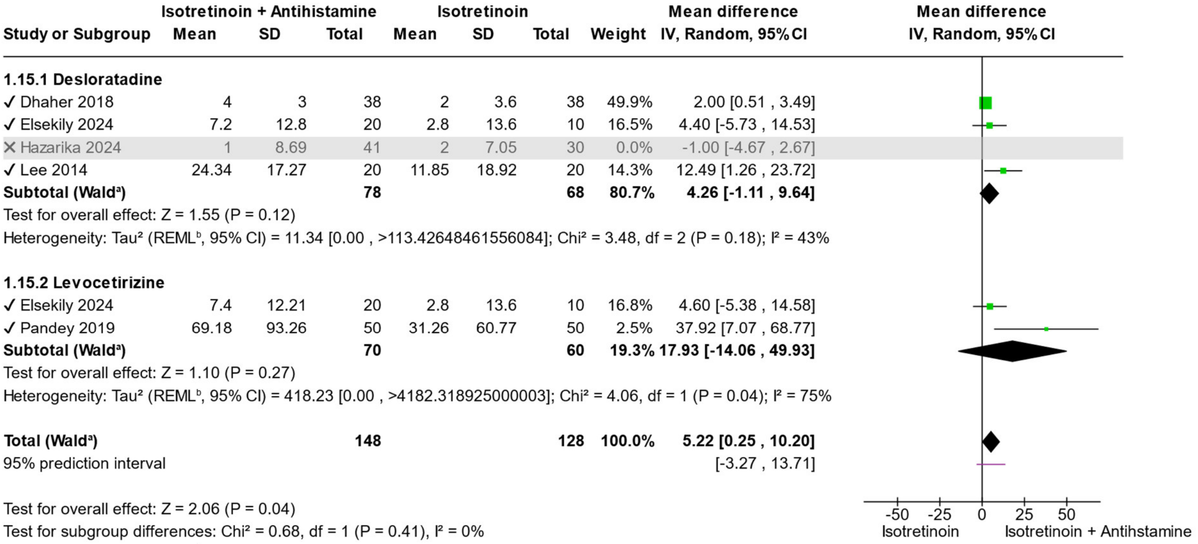

Test for overall effect: Z = 2.06 (P = 0.04)
Test for subgroup differences: Chi² = 0.68, df = 1 (P = 0.41), I² = 0%
Heterogeneity: Tau² (REML[b], 95% CI) = 12.34 [0.00 , >123.35409965526816]; Chi² = 8.80, df = 4 (P = 0.07); I² = 40%

Footnotes
[a]CI calculated by Wald-type method.
[b]Tau² calculated by Restricted Maximum-Likelihood method.

Figure S21. Non-inflammatory lesions count mean change from baseline till week 4

Study or Subgroup	Isotretinoin + Antihistamine Mean	SD	Total	Isotretinoin Mean	SD	Total	Weight	Mean difference IV, Random, 95% CI
**1.16.1 Desloratadine**								
Asilian 2015	5.79	5.69	28	4.57	6.59	28	13.9%	1.22 [-2.00 , 4.44]
Dhaher 2018	7	2.65	38	6	3.6	38	71.7%	1.00 [-0.42 , 2.42]
Hazarika 2024	3	8.43	41	4.5	5.88	30	13.1%	-1.50 [-4.83 , 1.83]
Lee 2014	30.77	17.27	20	22.9	19.02	20	1.1%	7.87 [-3.39 , 19.13]
**Subtotal (Wald[a])**			**127**			**116**	**99.8%**	**0.78 [-0.42 , 1.99]**
Test for overall effect: Z = 1.27 (P = 0.20)								
Heterogeneity: Tau² (REML[b], 95% CI) = 0.00 [0.00 , >100]; Chi² = 3.49, df = 3 (P = 0.32); I² = 0%								
**1.16.2 Levocetirizine**								
Pandey 2019	88.08	93.7	50	52.31	58.58	50	0.2%	35.77 [5.14 , 66.40]
**Subtotal**			**50**			**50**	**0.2%**	**35.77 [5.14 , 66.40]**
Test for overall effect: Z = 2.29 (P = 0.02)								
Heterogeneity: Not applicable								
**Total (Wald[a])**			**177**			**166**	**100.0%**	**0.84 [-0.37 , 2.04]**
95% prediction interval								[-0.37 , 2.04]

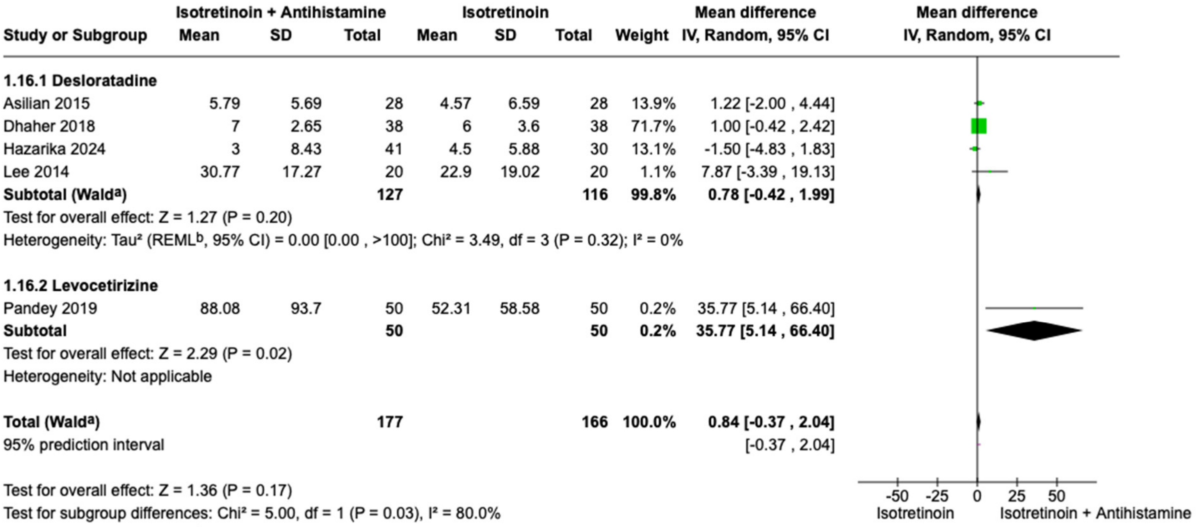

Test for overall effect: Z = 1.36 (P = 0.17)
Test for subgroup differences: Chi² = 5.00, df = 1 (P = 0.03), I² = 80.0%
Heterogeneity: Tau² (REML[b], 95% CI) = 0.00 [0.00 , >100]; Chi² = 8.49, df = 4 (P = 0.08); I² = 0%

Footnotes
[a]CI calculated by Wald-type method.
[b]Tau² calculated by Restricted Maximum-Likelihood method.

Figure S22. Non-inflammatory lesions count mean change from baseline till week 8

Study or Subgroup	Isotretinoin + Antihistamine Mean	SD	Total	Isotretinoin Mean	SD	Total	Weight	Mean difference IV, Random, 95% CI
**1.17.1 Desloratadine**								
✓ Dhaher 2018	10	2.65	38	7	3.46	38	94.6%	3.00 [1.61 , 4.39]
✓ Elsekily 2024	17.2	13.26	20	14.5	12.93	10	1.9%	2.70 [-7.20 , 12.60]
✕ Hazarika 2024	7	8.6	41	6.5	6	30	0.0%	0.50 [-2.90 , 3.90]
✓ Lee 2014	34.08	17.43	20	24.14	18.83	20	1.4%	9.94 [-1.31 , 21.19]
**Subtotal (Wald[a])**			**78**			**68**	**97.9%**	**3.10 [1.73 , 4.46]**
Test for overall effect: Z = 4.45 (P < 0.00001)								
Heterogeneity: Tau² (REML[b], 95% CI) = 0.00 [0.00 , >100]; Chi² = 1.45, df = 2 (P = 0.48); I² = 0%								
**1.17.2 Levocetirizine**								
✓ Elsekily 2024	17.5	12.48	20	14.5	12.93	10	1.9%	3.00 [-6.70 , 12.70]
✓ Pandey 2019	94.25	94.27	50	57.91	58.33	50	0.2%	36.34 [5.61 , 67.07]
**Subtotal (Wald[a])**			**70**			**60**	**2.1%**	**16.35 [-15.67 , 48.37]**
Test for overall effect: Z = 1.00 (P = 0.32)								
Heterogeneity: Tau² (REML[b], 95% CI) = 420.63 [0.00 , >4206.325770000004]; Chi² = 4.11, df = 1 (P = 0.04); I² = 76%								
**Total (Wald[a])**			**148**			**128**	**100.0%**	**3.16 [1.81 , 4.51]**
95% prediction interval								[1.81 , 4.51]

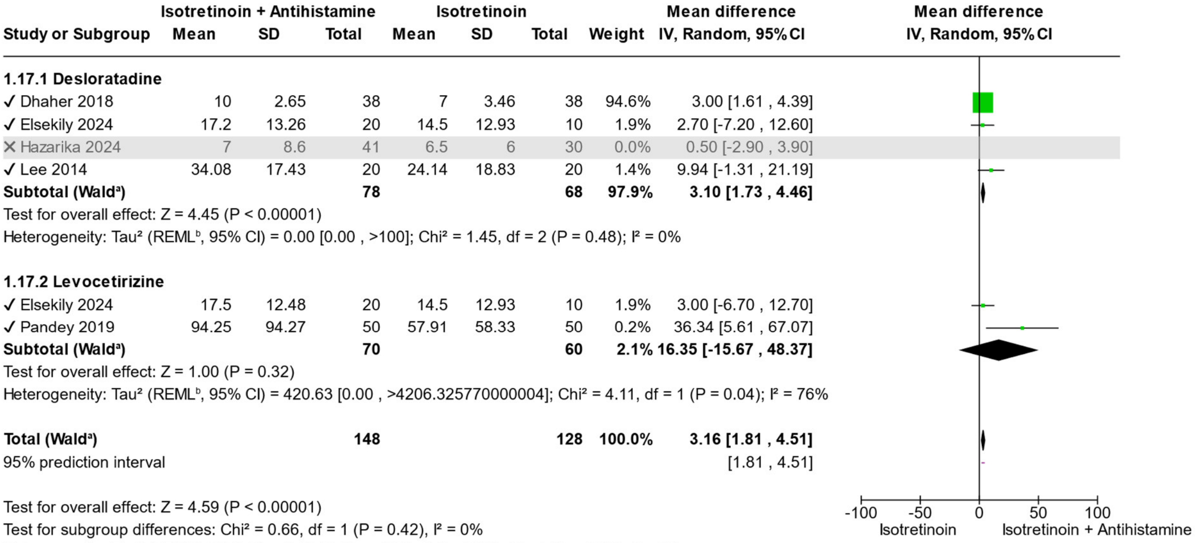

Test for overall effect: Z = 4.59 (P < 0.00001)
Test for subgroup differences: Chi² = 0.66, df = 1 (P = 0.42), I² = 0%
Heterogeneity: Tau² (REML[b], 95% CI) = 0.00 [0.00 , >100]; Chi² = 5.94, df = 4 (P = 0.20); I² = 0%

Footnotes
[a]CI calculated by Wald-type method.
[b]Tau² calculated by Restricted Maximum-Likelihood method.

Figure S23. Non-inflammatory lesions count mean change from baseline till week 12

Study or Subgroup	Isotretinoin + Antihistamine Mean	SD	Total	Isotretinoin Mean	SD	Total	Weight	Mean difference IV, Random, 95% CI
**1.17.1 Desloratadine**								
✕ Dhaher 2018	10	2.65	38	7	3.46	38	0.0%	3.00 [1.61 , 4.39]
✓ Elsekily 2024	17.2	13.26	20	14.5	12.93	10	15.6%	2.70 [-7.20 , 12.60]
✓ Hazarika 2024	7	8.6	41	6.5	6	30	53.8%	0.50 [-2.90 , 3.90]
✓ Lee 2014	34.08	17.43	20	24.14	18.83	20	12.6%	9.94 [-1.31 , 21.19]
**Subtotal (Wald[a])**			**81**			**60**	**82.0%**	**2.22 [-2.31 , 6.76]**
Test for overall effect: Z = 0.96 (P = 0.34)								
Heterogeneity: Tau² (REML[b], 95% CI) = 4.83 [0.00 , >100]; Chi² = 2.55, df = 2 (P = 0.28); I² = 23%								
**1.17.2 Levocetirizine**								
✓ Elsekily 2024	17.5	12.48	20	14.5	12.93	10	16.1%	3.00 [-6.70 , 12.70]
✓ Pandey 2019	94.25	94.27	50	57.91	58.33	50	2.0%	36.34 [5.61 , 67.07]
**Subtotal (Wald[a])**			**70**			**60**	**18.0%**	**16.35 [-15.67 , 48.37]**
Test for overall effect: Z = 1.00 (P = 0.32)								
Heterogeneity: Tau² (REML[b], 95% CI) = 420.63 [0.00 , >4206.325770000004]; Chi² = 4.11, df = 1 (P = 0.04); I² = 76%								
**Total (Wald[a])**			**151**			**120**	**100.0%**	**3.14 [-1.22 , 7.49]**
95% prediction interval								[-3.39 , 9.66]

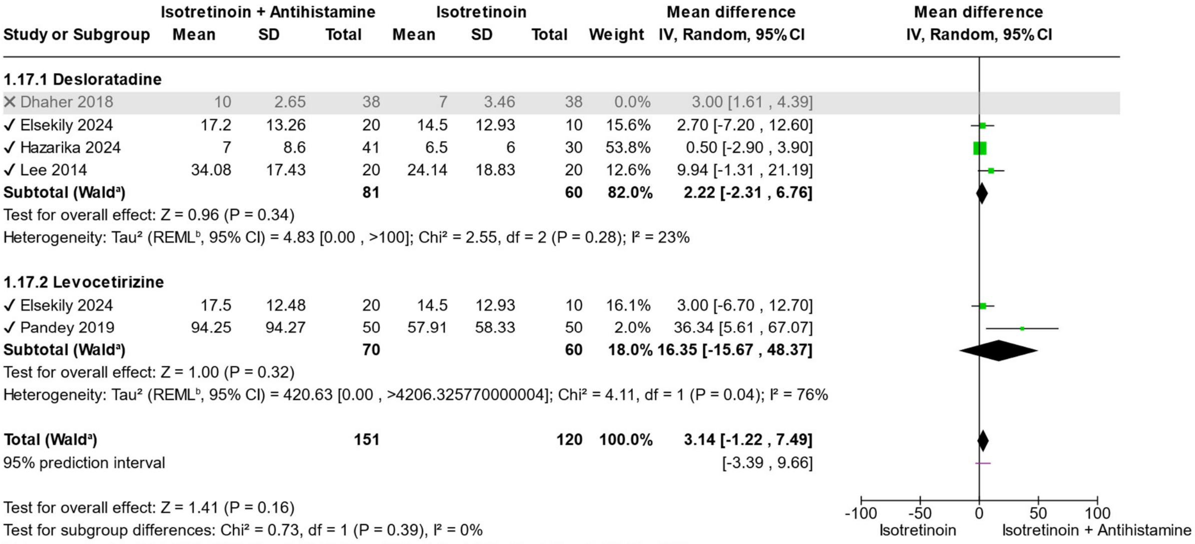

Test for overall effect: Z = 1.41 (P = 0.16)
Test for subgroup differences: Chi² = 0.73, df = 1 (P = 0.39), I² = 0%
Heterogeneity: Tau² (REML[b], 95% CI) = 6.15 [0.00 , >100]; Chi² = 7.52, df = 4 (P = 0.11); I² = 23%

Footnotes
[a]CI calculated by Wald-type method.
[b]Tau² calculated by Restricted Maximum-Likelihood method.

Figure S24. Non-inflammatory lesions count mean change from baseline till week 12

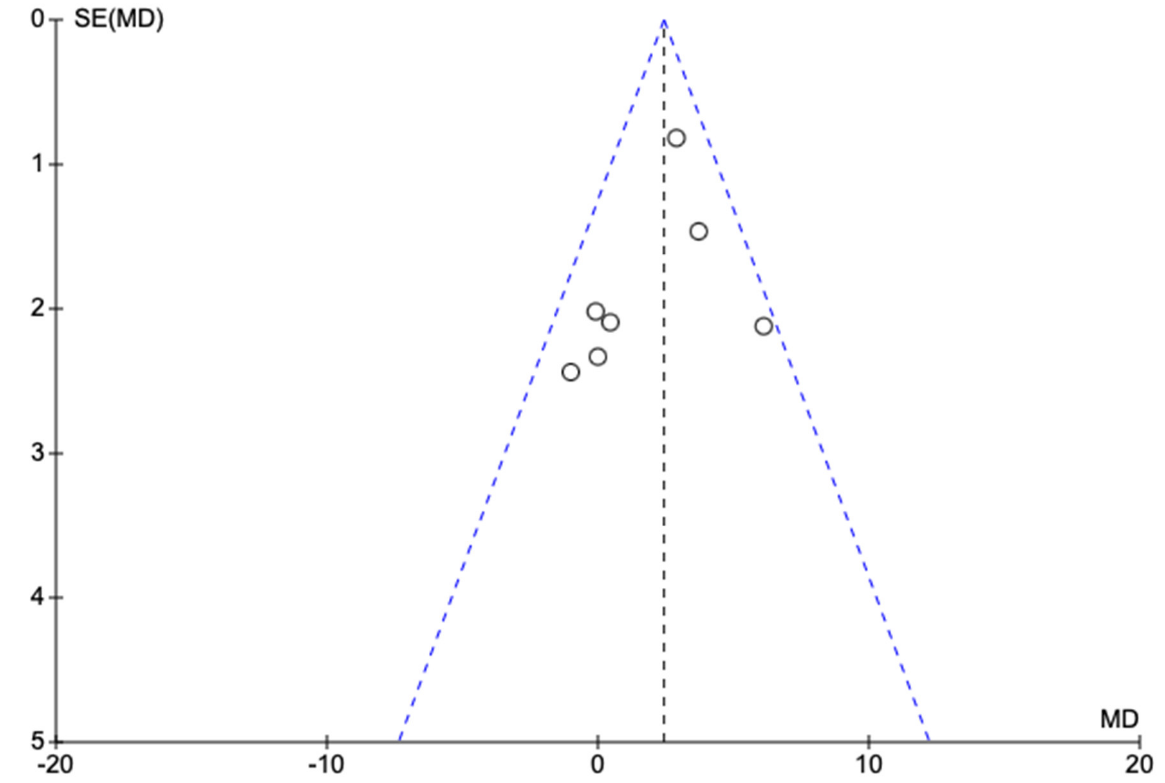

Figure S25. GAGS mean change from baseline till week 12 – funnel plot

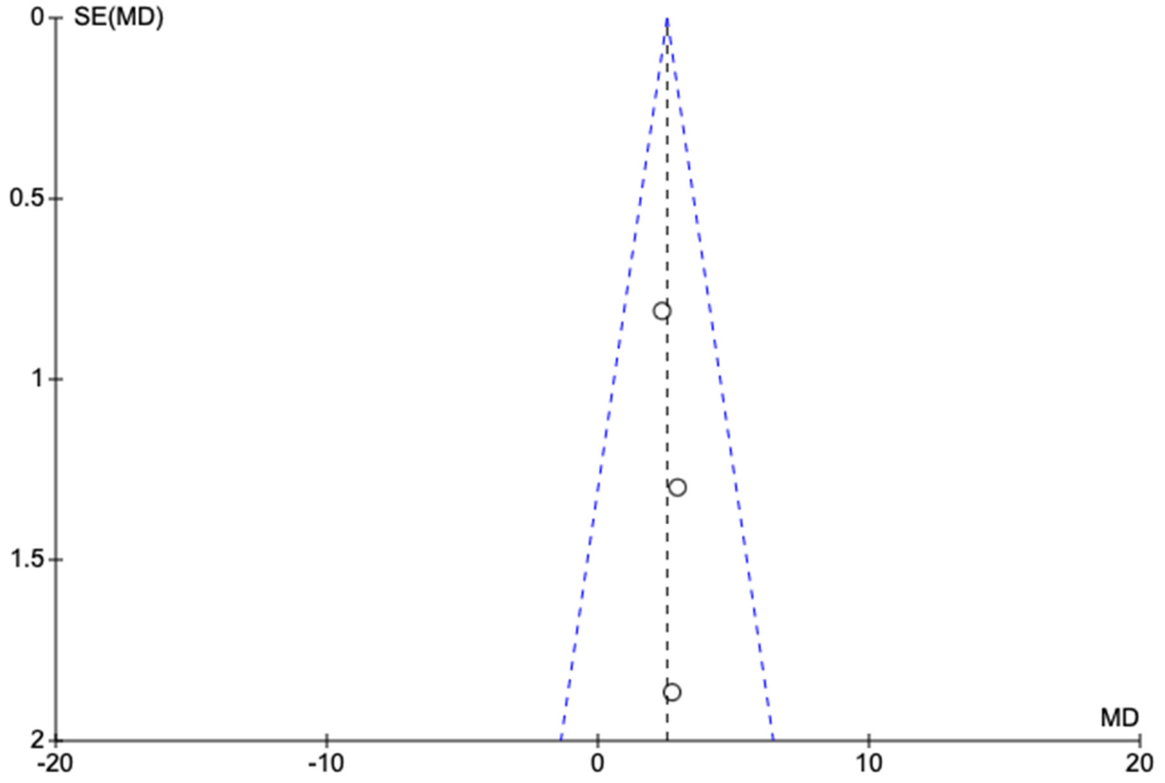

Figure S26. GAGS mean change from baseline till week 16 – funnel plot

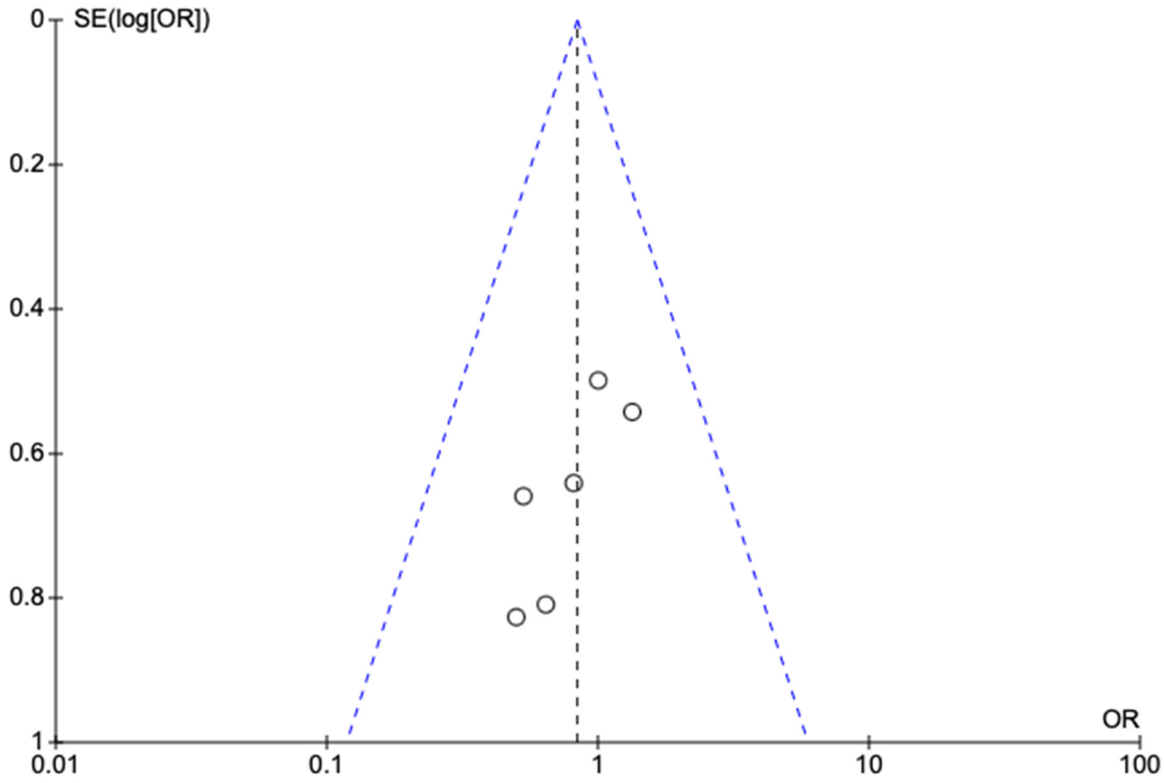

Figure S27. Xerosis – funnel plot
